# Sympathetic nerve activity and response to physiological stress in Takotsubo syndrome

**DOI:** 10.1007/s10286-024-01082-9

**Published:** 2024-11-15

**Authors:** Christina Ekenbäck, Jonas Persson, Per Tornvall, Lena Forsberg, Jonas Spaak

**Affiliations:** 1 Division of Cardiovascular Medicine, Department of Clinical Sciences, Danderyd Hospital, Karolinska Institutet, Stockholm, Sweden; 2 Department of Cardiology, Danderyd University Hospital, Stockholm, Sweden; 3 Department of Clinical Science and Education, Södersjukhuset, Karolinska Institutet, Stockholm, Sweden; 4 Department of Molecular Medicine and Surgery, Karolinska Institutet, Stockholm, Sweden; 5 Department of Clinical Physiology, Karolinska University Hospital, Stockholm, Sweden

**Keywords:** Takotsubo syndrome, Microneurography, Muscle sympathetic nerve activity, Iodine 123-metaiodobenzylguanidine scintigraphy, Cardiac sympathetic activity

## Abstract

**Purpose:**

The prevailing hypothesis posits that Takotsubo syndrome (TTS) is caused by massive sympathetic activation, yet supporting evidence remains inconsistent. The objectives of the present study were to determine whether sympathetic activity and reactivity are enhanced in the recovery phase of TTS, and to evaluate the effect of selective β1-receptor blockade on sympathetic reactivity.

**Methods:**

We conducted a case–control study that included 18 female patients with TTS and 13 age- and sex-matched controls. Muscle sympathetic nerve activity was measured through microneurography of the peroneal nerve at rest and during the cold pressor test. In the TTS group, recordings were repeated after randomisation to intravenous metoprolol or placebo. In 10 TTS patients, cardiac sympathetic activity was assessed using iodine 123-metaiodobenzylguanidine scintigraphy. Blood samples were collected during hospitalisation.

**Results:**

Microneurography was performed a median of 27.5 days after patient admission. There were no significant differences in burst incidence, burst frequency, burst height or burst area between the TTS patients and the controls at rest, during stress or after administration of intravenous metoprolol. Iodine 123-metaiodobenzylguanidine scintigraphy was performed a median of 12.5 days after admission, revealing decreased early 1.54 ± 0.13 and late 1.40 ± 0.13 heart-to-mediastinum ratios, and an increased washout rate of 41.8 ± 12.1%. Catecholamine metabolites were comparable between the study groups.

**Conclusion:**

General sympathetic hyperactivity or hyperreactivity unlikely contributes to TTS, as catecholamine levels and muscle sympathetic nerve activity at rest and during stress were similar between the TTS patients and the controls. As scintigraphy showed increased cardiac sympathetic activity, a pathological cardiac adrenergic response and vulnerability to sympathetic activation may be crucial for the development of the syndrome.

## Introduction

Takotsubo syndrome (TTS) is a condition characterised by acute, reversible heart failure and a clinical presentation mimicking myocardial infarction. Notably, coronary angiography reveals no stenoses corresponding to the regional left ventricular dysfunction. A small proportion of patients with acute coronary syndrome are eventually diagnosed with TTS, predominantly postmenopausal women [1, 2]. Despite its reversibility, the prognosis of TTS is no better than that of myocardial infarction [3, 4], and to date no studies on treatment have been published.

The pathophysiological mechanisms of TTS remain elusive. Given its association with mental and physical stress, central sympathetic activation has been proposed as a key mediator [5]. Stress activates the sympathetic nervous system, which regulates heart rate and contractility through catecholamines released from the adrenal glands and cardiac sympathetic nerves. A surge in catecholamines is thought to induce TTS through mechanisms such as direct catecholamine toxicity, negative inotropic signalling, coronary vasoconstriction, myocardial oedema, altered myocardial metabolism, inflammation and relative ischaemia due to increased cardiac workload [6–10]. In support of the sympathetic theory, studies have reported elevated levels of plasma catecholamines, particularly epinephrine, in the acute phase of TTS [5, 11, 12], as well as increased levels of norepinephrine (NE) in the coronary sinus of TTS patients [13]. However, conflicting results have been reported, and it has also been argued that increased catecholamine levels could be a consequence rather than the cause of acute heart failure in TTS [13–15]. Results from direct measurements of muscle sympathetic nerve activity (MSNA) in TTS patients using microneurography are inconsistent [16–18], although iodine 123-metaiodobenzylguanidine (^123^I-mIBG) scintigraphy studies consistently show decreased myocardial ^123^I-mIBG uptake and increased washout rate (WR), indicating an increased cardiac specific sympathetic activation in TTS [11, 12, 19–22].

Against this background, we aimed to determine whether sympathetic activity and reactivity are enhanced in the early recovery phase of TTS. Since TTS patients are commonly prescribed beta-blockers (β-blockers), we also aimed to evaluate the effect of selective β1-receptor blockade on sympathetic reactivity in TTS.

## Methods

### Study design and study groups

The Sympathetic And vascular Function in Takotsubo syndrome (SAFT) study, conducted as a case–control study at Danderyd University Hospital, Stockholm, Sweden, enrolled consecutive female TTS patients aged 40 to 80 years as previously described [23].

Twenty-seven TTS patients were enrolled in the SAFT study between April 2016 and January 2021, and these patients were invited to undergo a follow-up microneurography as well as ^123^I-mIBG scintigraphy according to the study protocol. Sixteen female volunteers without prior cardiovascular events, active cancer, or inflammatory disease, and matched for age on the group level, were recruited through advertising on social media to serve as healthy controls for microneurography. Blood samples were drawn at inclusion during the index hospitalisation event for TTS patients and in the morning before microneurography for the controls.

### Microneurography

Microneurography enables direct and dynamic recording of MSNA in efferent postganglionic unmyelinated C-fibres of a peripheral nerve in awake humans [24], with a correlation to cardiac sympathetic outflow measured invasively [25]. MSNA recordings were conducted during the morning in a tranquil laboratory environment, with study subjects instructed to cease β-blocker use for 24 h and restricted to a caffeine-free, light breakfast. In a comfortable supine position, peroneal nerve localisation and tungsten microelectrode insertion were performed as previously described [26]. A sudden vocal arousal was performed to differentiate MSNA from skin sympathetic nerve activity. Baseline MSNA was recorded for 10 min at a 10 kHz sampling rate (PowerLab NeuroAmp EX neural amplifier device and LabChart 8 software; ADInstruments, Bella Vista, Australia), followed by MSNA recording during stress, induced by a cold pressor test [27], with levels determined between 30 and 60 and between 60 and 90 s of stress. MSNA recordings were repeated in TTS patients after randomisation in a 1:1 fashion to selective β1-receptor blockade (metoprolol) or placebo (saline). Metoprolol (1 mg/ml) was administered as intravenously injected doses of 2.5 to 5 mg at a time until systolic blood pressure (SBP) < 100 mmHg or heart rate (HR) < 60/min. The patients, but not the investigators, were blinded to the randomisation.

Raw neurograms were integrated using a time-constant of 0.2 s, rectified and time-shifted to display pulse-synchronous bursts. Bursts were identified and characterised using the software feature Peak Analysis and expressed as burst incidence (bursts/100 RR-intervals), burst frequency (bursts/min), burst height (μV) and burst area (μVs). Study subjects were monitored with standard electrocardiography (ECG) chest leads recording HR (beats/min), and with the volume clamp method (Finapres, Ohmeda, Madison, WI, USA) recording beat-to-beat SBP and diastolic blood pressure (DBP) (mmHg) continuously. Mean arterial pressure (MAP) (mmHg) was calculated as DBP + (SBP – DBP)/3. Intermittent BP recordings were also obtained using a calibrated sphygmomanometer on the upper arm.

### ^123^I-mIBG Scintigraphy

Iodine 123-metaiodobenzylguanidine is a gamma-emitting NE analogue used to assess the integrity and activity of cardiac sympathetic innervation [11]. ^123^I-mIBG scintigraphy was performed in TTS patients after overnight fasting, thyroid blockade, and 30 min of strictly enforced supine rest. A dose of 370 MBq of ^123^I-mIBG (74 MBq/ml; Mallinckrodt Netherlands, 's-Hertogenbosch, the Netherlands) was administered with 20 mL saline. All images were acquired using a 128 × 128 matrix with a dual detector gamma camera (Infinia II; GE, Waukesha, WI, USA) equipped with a low-energy, high-resolution collimator. Planar imaging was initiated 30 min after injection for early images and 240 min for late images. The planar ^123^I-mIBG images were analysed by a region-of-interest (ROI) technique to obtain semiquantitative parameters for tracer distribution.

The ^123^I-mIBG count densities of the heart (H) and the mediastinum (M) were calculated from the 30- and 240-min images. The heart-to-mediastinum (H/M) ratios of ^123^I-mIBG uptake at 30 min (early H/M) and at 240 min (late H/M) together with the washout rate (WR) (%) from the myocardium was calculated as previously reported [28]. A mean (± standard deviation [SD] H/M ratio of ≥ 2.2 ± 0.3 and a mean WR of ≤ 10 ± 9% were considered to be normal [29].

### Statistics

Continuous variables were reported as the mean ± SD or as the median with interquartile range (IQR), with normality assessed using the Shapiro–Wilk test. Dichotomous variables were reported as frequency and percentage. There were no missing data for the primary variables, and no imputations were performed. For comparison of continuous variables between groups, the Student´s t-test was used for normally distributed data and the Mann Whitney U-test was used for skewed data. The Pearson Chi-square (*χ*^2^) and Fisher´s exact two-sided tests were used for comparison of dichotomous variables between groups. Repeated measures one-way and two-way ANOVA with the Tukey HSD post hoc test were used to analyse time-dependant differences in variables within and between study groups. Associations between continuous variables were evaluated with linear regression and the Spearman rank test. A two-sided *P* value < 0.05 indicated statistical significance. STATA version 13.1 (StataCorp, College Station, TX, USA) was used for statistical analyses.

## Results

### Study groups

Of the 27 TTS patients enrolled in the SAFT study between April 2016 and January 2021, six declined to undergo a follow-up microneurography and in an additional three patients the signal:noise ratio was too low to be analysed. Thus, 18 TTS patients remained for the subsequent analyses. Of the 16 age- and sex-matched controls, in three individuals it was not possible to acquire a noise-free MSNA signal, leaving 13 controls for analyses. The study groups were overall comparable in terms of age, menopausal status, cardiovascular risk factors, psychiatric and inflammatory disorders, body mass index and levels of haemoglobin and creatinine. None of the TTS patients was on antipsychotic or anxiolytic medication during the study, compared to one individual in the control group. Levels of oestradiol were postmenopausal in both groups (reference level < 37 pmol/L), but higher in TTS patients than controls. N-terminal pro-brain natriuretic peptide (NT-proBNP) was elevated to a greater extent than troponin T amongst TTS patients, whereas biomarker levels were within normal ranges in the controls. Levels of plasma catecholamine metabolites did not differ significantly between the study groups, although the median methoxyepinephrine level was slightly elevated in TTS patients (reference level < 60 ng/L) (Table 1).

**Table 1:** Baseline characteristics between patients with Takotsubo syndrome and controls

Variable	Takotsubo syndrome group (*n* = 18)	Control group (*n* = 13)	*P* value
Female	18 (100)	13 (100)	
Age, years	66.8 ± 9.91	66.5 ± 6.35	0.940
Post-menopausal	16 (89)	13 (100)	0.214
Heredity for CAD	5 (28)	2 (15)	0.667
Hypertension	6 (33)	1 (8)	0.191
Hyperlipidaemia	3 (17)	1 (8)	0.621
Diabetes mellitus	2 (11)	0 (0)	0.497
Active smoker	2 (11)	0 (0)	0.497
Former smoker	12 (67)	6 (46)	0.253
Psychiatric disorder	4 (22)	1 (8)	0.368
Inflammatory disorder	4 (22)	1 (8)	0.368
BMI, kg/m^2^	23.9 ± 3.84	24.0 ± 4.47	0.931
Haemoglobin, g/L	131 (125–145)	138 (127–139)	0.501
Creatinine, μmol/L	62.7 ± 7.23	65.6 ± 9.17	0.368
Oestradiol, pmol/L	17 (12–38)	11 (10–14)	0.029
Troponin T, ng/L	262 (144–555)	7 (7–7)	< 0.001
NT-proBNP, ng/L	2440 (1200–7350)	74 (43–129)	< 0.001
Methoxyepinephrine, ng/L	63.7 (34.0–92.7)	40.8 (36.7–45.7)	0.052
Methoxynorepinephrine, ng/L	104 (77.1–124)	105 (78.2–137)	0.548

Values in table are reported as numbers (%), the mean ± standard deviation or the median with the interquartile range in parentheses, as appropriate

*BMI* body mass index, *CAD* coronary artery disease, *NT−proBNP* N−terminal pro B−type natriuretic peptide

### Microneurography

**Fig. 1:**
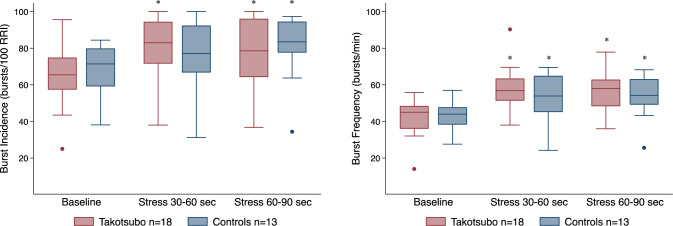
Box plots of burst incidence (bursts/100 RRI) (left panel) and burst frequency (bursts/min) (right panel) between patients with Takotsubo (*n* = 18) and controls (*n* = 13) at baseline, and at 30 to 60 and 60 to 90 s of stress induced by cold pressor test. Asterisk indicates a significant change from baseline (*P < 0.01). *RRI* RR intervals

**Fig. 2:**
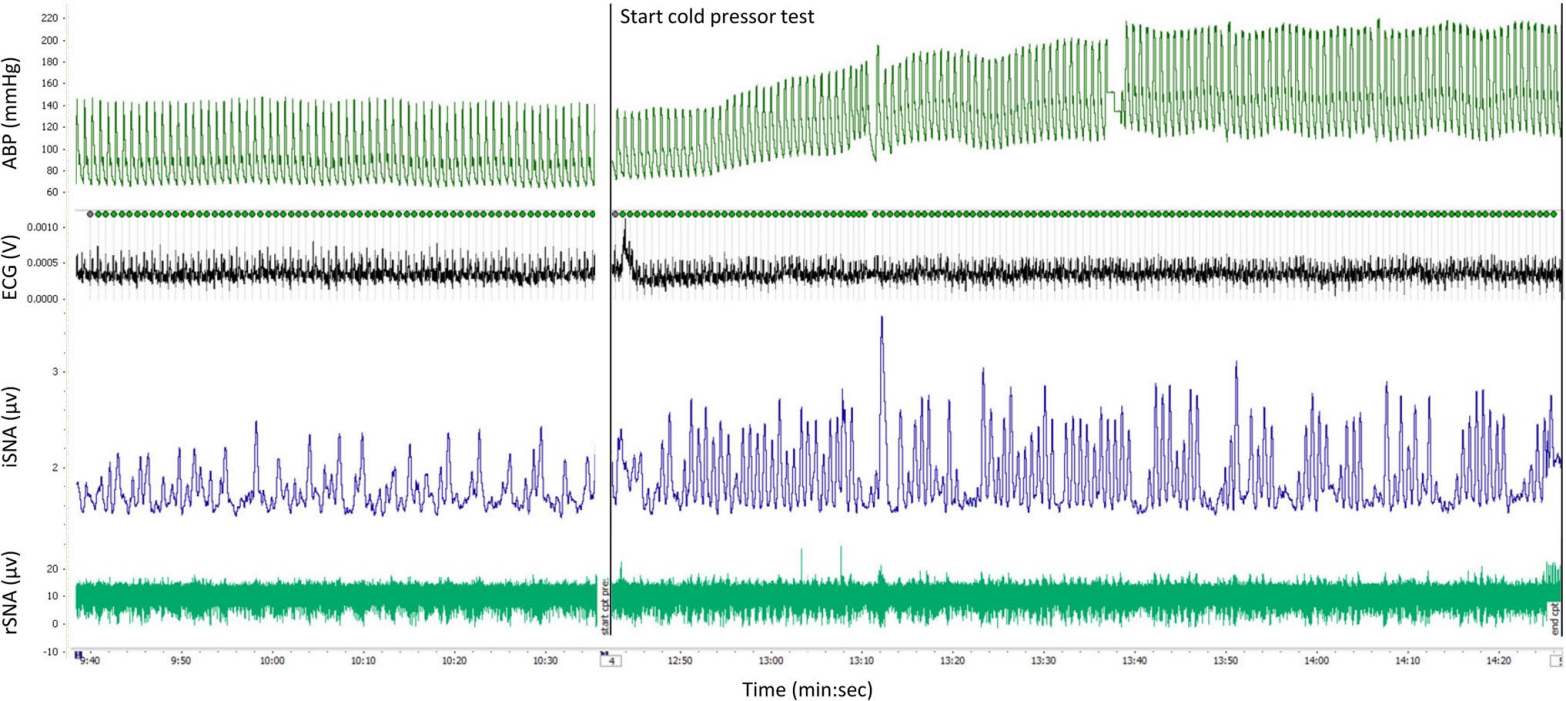
An example of a typical muscle sympathetic nerve activity (MSNA) recording. *ABP* Arterial blood pressure, *ECG*   electrocardiogram, *iSNA* integrated sympathetic activity, *rSNA*   raw sympathetic activity.

Patients with TTS underwent microneurography a median of 27.5 (IQR 22–31) days after admission. All patients and controls were studied during sinus rhythm. Levels of burst incidence, burst frequency, burst height, burst area, SBP and MAP did not differ between TTS patients and controls at baseline, and at 30 to 60 s or at 60 to 90 s of stress. All measures of MSNA and haemodynamic parameters increased significantly during stress in both groups, except for HR in the controls (Table 2). Box plots of burst incidence and burst frequency between TTS patients and controls are illustrated in Fig. 1. Burst incidence and burst frequency were not associated with time from admission to MSNA recording (*R*_*s*_ = - 0.144; *P* = 0.582 and *R*_*s*_ = - 0.016; *P* = 0.952, respectively). An example of a typical MSNA recording is shown in Fig. 2. In the repeated MSNA recordings in TTS patients, levels of MSNA and haemodynamic parameters did not differ at baseline or during stress between patients randomised to metoprolol (*n* = 9) or placebo (*n* = 9) (Table 3).

**Table 2:** Muscle sympathetic nerve activity and haemodynamic parameters at baseline, and at 30 to 60 and 60 to 90 s of stress between patients with Takotsubo syndrome and controls

Muscle sympathetic nerve activity and haemodynamic parameters	Baseline		Stress: 30 to 60 s				Stress: 60 to 90 s				*P-value*^b^
	Takotsubo syndrome group (*n* = 18)	Control group (*n* = 13)	Takotsubo syndrome group (*n* = 18)	*P-value*^a^	Control group (*n* = 13)	*P-value*^a^	Takotsubo syndrome group (*n* = 18)	*P-value*^a^	Control group (*n* = 13)	*P-*value^a^	
Burst incidence, bursts/100 RRI	65.4 (57.4–74.8)	71.4 (59.1–79.9)	83.0 (71.6–94.4)	0.000	77.1 (66.8–92.4)	0.064	78.6 (64.2–96.1)	0.001	83.5 (77.6–94.5)	0.009	0.433
Burst frequency, bursts/min	41.9 ± 10.2	43.1 ± 8.02	58.0 ± 12.0	0.000	52.8 ± 12.9	0.004	56.8 ± 11.3	0.000	53.7 ± 11.4	0.001	0.128
Burst height, μV	0.29 (0.21–0.43)	0.33 (0.26–0.56)	0.44 (0.37–0.54)	0.017	0.44 (0.31–0.89)	0.032	0.42 (0.38–0.76)	0.001	0.52 (0.32–0.90)	0.024	0.653
Burst area, μVs	0.09 ± 0.04	0.13 ± 0.08	0.14 ± 0.06	0.004	0.17 ± 0.10	0.017	0.15 ± 0.08	0.001	0.16 ± 0.09	0.038	0.483
HR, beats/min	63 ± 12	64 ± 7	73 ± 16	0.000	69 ± 9	0.389	74 ± 16	0.000	67 ± 9	0.771	0.025
SBP, mmHg	149 ± 24	141 ± 17	186 ± 29	0.000	179 ± 22	0.000	191 ± 34	0.000	183 ± 24	0.000	0.972
MAP, mmHg	94 ± 16	94 ± 11	115 ± 25	0.000	114 ± 14	0.000	120 ± 27	0.000	116 ± 16	0.000	0.832

*HR* Heart rate, *MAP* mean arterial pressure, *RRI* RR interval, *SBP* systolic blood pressure

Values are reported as the mean ± standard deviation or as the median with the interquartile range in parentheses, as appropriate

^a^Post−hoc* P*−values for change from baseline

^b^Two−way analysis of variance* P*-values between Takotsubo patients and controls

**Table 3:** Muscle sympathetic nerve activity and haemodynamic parameters at second baseline, and at 30 to 60 and 60 to 90 s of stress between patients with Takotsubo syndrome randomised to metoprolol or placebo

Muscle sympathetic nerve activity and haemodynamic parameters	Baseline		Stress: 30 to 60 s		Stress: 60 to 90 s		*P*-value^a^
	Metoprolol (*n* = 9)	Placebo (*n* = 9)	Metoprolol (*n* = 9)	Placebo (*n* = 9)	Metoprolol (*n* = 9)	Placebo (*n* = 9)	
Burst incidence, bursts/100 RRI	73.7 (58.9–90.8)	72.0 (52.0–83.0)	98.2 (74.4–100)	88.2 (49.9–96.2)	94.2 (84.8–97.2)	92.6 (67.0–94.0)	0.308
Burst frequency, bursts/min	41.4 ± 12.5	38.7 ± 8.76	58.5 ± 11.2	51.8 ± 9.98	59.1 ± 10.6	58.2 ± 10.0	0.511
Burst height, μV	0.39 (0.16–0.40)	0.32 (0.25–0.39)	0.42 (0.19–0.49)	0.45 (0.42–0.64)	0.39 (0.18–0.45)	0.35 (0.31–0.58)	0.671
Burst area, μVs	0.09 ± 0.04	0.09 ± 0.02	0.13 ± 0.09	0.16 ± 0.04	0.12 ± 0.06	0.12 ± 0.03	0.534
HR, beats/min	58 ± 8	60 ± 12	65 ± 11	73 ± 18	67 ± 12	76 ± 22	0.154
SBP, mmHg	158 ± 18	152 ± 15	181 ± 26	190 ± 24	191 ± 35	193 ± 19	0.237
MAP, mmHg	101 ± 17	96 ± 8	118 ± 26	121 ± 19	125 ± 30	121 ± 17	0.318

Values are reported as the mean ± standard deviation or as the median with the interquartile range in parentheses, as appropriate

*HR* Heart rate, *MAP* mean arterial pressure, *RRI* RR intervals, *SBP* systolic blood pressure

^a^Two−way analysis of variance* P*-values between Takotsubo patients and controls

### ^123^I-mIBG scintigraphy

**Fig. 3:**
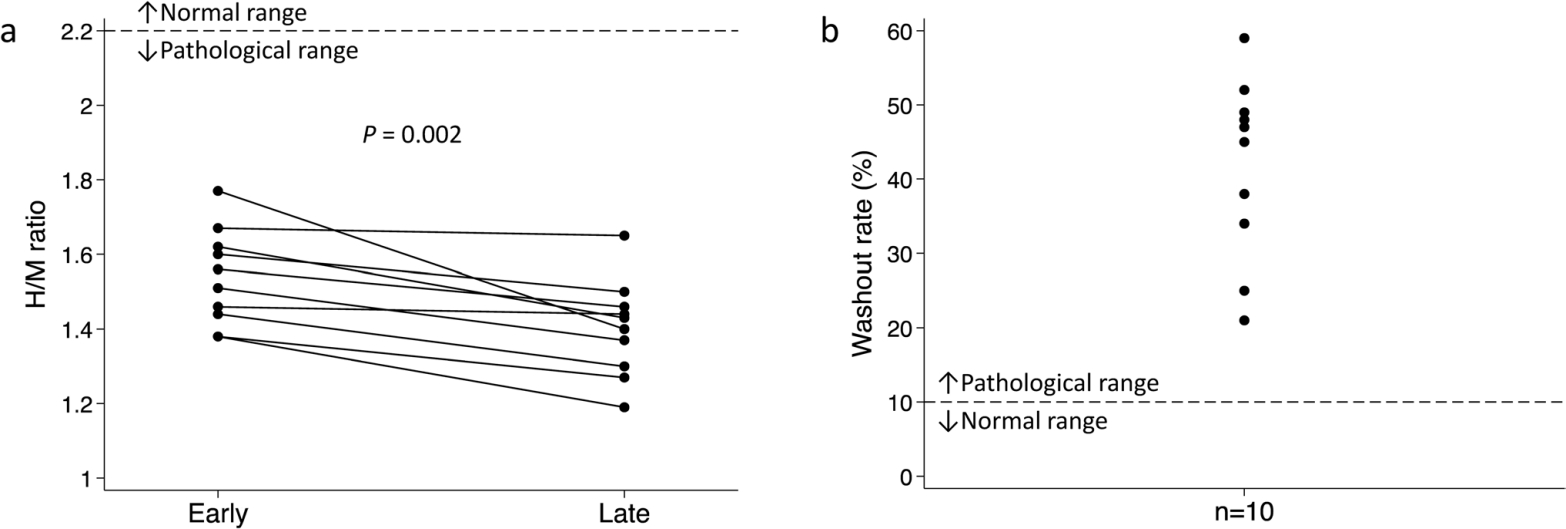
Results of ^123^I-mIBG scintigraphy for patients with Takotsubo syndrome (*n* = 10). **a** Early and late H/M ratios with normal mean shown as the dashed line. **b** Washout rate (%) with normal mean shown as the dashed line. *H/M* Heart-to-mediastinum, ^*123*^*I-mIBG* Iodine 123-metaiodobenzylguanidine

**Fig. 4:**
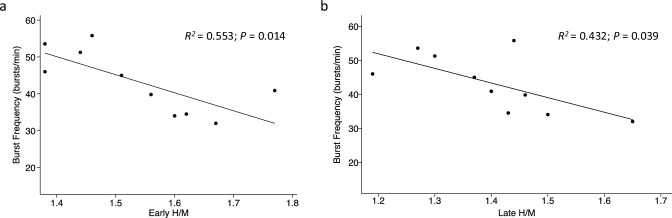
The relationship between burst frequency (bursts/min) and early H/M (**a**) and late H/M (**b**) in patients with Takotsubo syndrome (*n* = 10).* H/M* Heart-to-mediastinum ratio

Of the 18 TTS patients examined with microneurography, ten (56%) also underwent ^123^I-mIBG scintigraphy. ^123^I-mIBG scintigraphy was performed a median of 12.5 (IQR 10–15) days after hospital admission and revealed a lower-than-normal mean (± SD) early H/M (1.54 ± 0.13) and mean late H/M (1.40 ± 0.13), and a higher-than-normal mean WR (41.8 ± 12.1%) (*P* < 0.001). All TTS patients had decreased early and late H/M and increased WR. The H/M ratio differed significantly between early and late images (Fig. 3). Early and late H/M showed significant negative correlations to baseline burst frequency (*R*^2^ = 0.553, *P* = 0.014 and* R*^2^ = 0.432 *P* = 0.039, respectively) (Fig. 4). Neither H/M ratios nor WR correlated with baseline burst incidence (data not shown).

## Discussion

Based on the results of our study, we found no differences in MSNA at rest or during physiological stress between female patients in the early recovery phase of TTS and age- and sex-matched controls, and no effect of selective β1-receptor blockade on MSNA response to stress in TTS patients. In contrast, ^123^I-mIBG scintigraphy showed decreased H/M ratios and increased WR in all TTS patients, although catecholamine metabolites during hospitalisation were not significantly elevated.

### Main findings in relation to previous studies

In our study, baseline MSNA in TTS patients and controls was comparable to normal values of women in the same age range [30]. Vaccaro et al. [16] measured MSNA at rest in 13 female patients with TTS within 72 h of symptom onset, with an equally sized and matched cohort of patients with acutely decompensated chronic heart failure as controls. Although MSNA is commonly elevated in heart failure [31], burst incidence was even higher in their TTS cohort, and higher than that in our TTS patients in the recovery phase. Considering the reversible nature of TTS, the timing of MSNA assessment could explain the discrepancy between our study and that of Vaccaro et al. [16]. Sverrisdóttir et al. [17] found comparable MSNA levels at rest in 12 female TTS patients and matched healthy controls. However, burst incidence was significantly lower in the subgroup of TTS patients in the recovery phase (*n* = 7) than in controls, speculated to be a consequence of reflex widespread sympathetic inhibition following the excessive catecholamine release responsible for the development of TTS. Since burst incidence in controls was comparable between our studies, the discrepancy in burst incidence between our patients and their TTS patients in the recovery phase cannot fully be explained by different sample sizes or timing of measurement. MSNA response to stress in TTS has previously been evaluated in one small study that compared six patients with a history of TTS to matched controls [18]. In line with our results, these authors found no differences in MSNA between the groups at rest or in response to stress.

In accordance with our data, previous ^123^I-mIBG scintigraphy studies are supportive of increased cardiac sympathetic activity in TTS. Christensen et al. [11] compared ^123^I-mIBG scintigraphy in TTS patients with controls who had other diagnoses, such as myocarditis and aborted infarction. These authors found that TTS patients had lower late H/M and higher WR than controls in the subacute phase, but found no differences between the groups at follow-up after 4 months, indicating a gradual normalisation of cardiac sympathetic activity along with restoration of cardiac function [11]. Burgdorf et al. [19] showed that ^123^I-mIBG uptake was regionally reduced in the akinetic apical ventricular segments of TTS patients in the subacute phase, despite normal or only mildly impaired perfusion. Others have shown reduced ^123^I-mIBG uptake and impaired glucose metabolism in the hypokinetic, but well-perfused, left ventricular segments of TTS patients, with a progressive improvement at 6 months of follow-up [20]. In a study by Sestini et al. [21], regional ^123^I-mIBG uptake was impaired in congruence with contractility and perfusion of the left ventricle in the acute phase of TTS. Despite normalisation of contractility and perfusion within the first month, residual apical ^123^I-mIBG uptake defects were present in half of the patients after 2 years [21]. The relationship between sympathetic activity and cardiac function was explored in a study that included 90 TTS patients divided according to left ventricular recovery into a soon (< 1 month) and a late (> 1 month) group [22]. The late group was characterised by more enhanced sympathetic activity with higher catecholamine levels, higher WR and lower late H/M, as well as higher rates of in-hospital complications [22].

To the best our knowledge, our study is the first to evaluate both MSNA and cardiac sympathetic activity in the same TTS cohort. Other studies have previously shown that MSNA has a weak negative correlation with H/M ratios and a strong positive correlation with WR in patients with myocardial infarction [32], as well as in patients with left ventricular dysfunction and exercise intolerance [33], in line with our findings of correlations between burst frequency and H/M ratios also in TTS.

### Possible mechanisms and implications

In normal physiological conditions, catecholamines exert both inotropic and chronotropic effects by binding to cardiac β-adrenoreceptors (βAR). These receptors have a higher affinity for epinephrine than NE. Central sympathetic activation triggered by stress increases the adrenal secretion of catecholamines, mainly epinephrine, into the circulation. High doses of circulating epinephrine have been shown to induce a switch from β_2_AR G-protein-coupled stimulatory (β_2_AR-Gs) to inhibitory (β_2_AR-Gi) pathways, resulting in TTS-like cardiac depression in rats [34]. A basal to apical gradient of βAR density was proposed as an explanation to why the apical segments are particularly vulnerable to catecholamine surges [34]. Myocardial stunning in TTS is supposedly aggravated by catecholamine-induced increased cardiac workload, inflammation, coronary microvascular dysfunction and altered metabolism [7]. Central sympathetic stimulation also causes a local NE release from cardiac sympathetic nerves. With an intense or prolonged cardiac sympathetic activation, the NE concentration in the synaptic cleft increases due to a saturation of the NE reuptake monoamine transporter, as well as by an increased presynaptic βAR-stimulation by circulating catecholamines. Together these effects lead to a gradual depletion of presynaptic NE storage, which can be depicted by ^123^I-mIBG scintigraphy [19, 28, 29]. While the early H/M ratio reflects both pre- and postsynaptic NE uptake, the late H/M ratio primarily reflects the presynaptic uptake [19]. Somewhat simplified, the combination of reduced late H/M ratio and increased WR has usually been interpreted as an increased cardiac sympathetic activity, although a functional impairment of cardiac sympathetic nerve integrity or a structural cardiac denervation with decreased sympathetic neuronal density could give the same pattern. Due to the apical to basal gradient of cardiac sympathetic innervation, the basal hyperkinesia and left ventricular outflow tract obstruction commonly evident in TTS are speculated to be NE induced [11].

Although most people experience stressful events in life, only very few develop TTS which predominantly affects postmenopausal women. In a study on ovariectomised female rats, oestrogen replacement was shown to attenuate the development of stress-induced Takotsubo-like cardiac responses [35]. Oestrogen deprivation following menopause could make women prone to develop TTS through several mechanisms, such as increased sympathetic drive, altered cardiovascular responses to stress and/or endothelial dysfunction [36, 37]. Despite a pattern consistent with increased cardiac sympathetic activity indicated by ^123^I-mIBG scintigraphy, we found no alterations in MSNA and no excessive elevation of catecholamine metabolites in our female TTS cohort, as compared to controls. These results could be consistent with an increased sympathetic nerve activation selectively to the heart. However, both the shown congruence between MSNA and cardiac-specific sympathetic outflow [25], and the fact that TTS can also affect functionally denervated heart transplant patients [38], argue against this. Altogether, our results indicate a pathological cardiac response in TTS, causing an increased sensitivity to sympathetic activation that does not need to be excessive.

Despite the reversible nature of TTS, long-term mortality aligns with that for myocardial infarction [3, 4], and recurrency is not uncommon [3]. However, there are currently no evidence-based treatment options for TTS. TTS patients are commonly prescribed standard heart failure treatment such as cardioselective β-blockers and angiotensin-converting enzyme inhibitors, although the effect or potential harm of these agents has not been evaluated in any trials. In our study, selective β1-receptor blockade was safely administrated but had no effect on MSNA in TTS patients. This result might not be surprising, as β_2_AR stimulating pathways, rather than β_1_AR pathways, have been proposed to be involved in the pathogenesis of TTS [34]. Interestingly, Marfella et al. showed that α-lipoic acid (ALA) therapy, which restores sympatho-vagal alterations in diabetic cardiomyopathy, improved ^123^I-mIBG defect size in patients with TTS at 1 year of follow-up [12]. Targeting cardiac sympathetic dysregulation in TTS appears to be an appealing treatment option, although the prognostic value of ALA therapy is still to be proven.

### Strengths and limitations

To our knowledge, this is the largest prospective study to date measuring sympathetic nerve activity in TTS using microneurography. In this all-female cohort with a comparable age- and sex-matched control group, microneurography before and during stress provocation was performed in the early recovery phase in all patients. Additionally, cardiac sympathetic activity was evaluated using ^123^I-mIBG scintigraphy prior to microneurography, and catecholamine metabolites from the acute phase were analysed.

However, we acknowledge several limitations. First, microneurography was performed in the recovery phase of all TTS patients. Although we found no association between MSNA and the time from admission to hospital, we cannot rule out an altered MSNA level or reactivity in the acute phase of the syndrome. Second, in our study, stress was induced by the cold pressor test only, and mental stress may have induced a different MSNA response. Last, the small study size increases the risk for type II errors.

## Conclusions

Levels of catecholamine metabolites during the acute phase of TTS, as well as levels of MSNA at rest and during physiological stress in the recovery phase of TTS, do not significantly differ from those in matched controls. These findings challenge the prevailing theory that TTS is caused by a massive general sympathetic activation. Together with our scintigraphy findings of decreased myocardial ^123^I-mIBG uptake and increased WR, our results imply that a pathological cardiac adrenergic response and vulnerability to sympathetic activation may be crucial for the development of the syndrome.

## Data Availability

Data may be shared pending relevant Ethical approval.
